# Genetic Architecture of Skin and Eye Color in an African-European Admixed Population

**DOI:** 10.1371/journal.pgen.1003372

**Published:** 2013-03-21

**Authors:** Sandra Beleza, Nicholas A. Johnson, Sophie I. Candille, Devin M. Absher, Marc A. Coram, Jailson Lopes, Joana Campos, Isabel Inês Araújo, Tovi M. Anderson, Bjarni J. Vilhjálmsson, Magnus Nordborg, António Correia e Silva, Mark D. Shriver, Jorge Rocha, Gregory S. Barsh, Hua Tang

**Affiliations:** 1 Department of Genetics, Stanford University School of Medicine, Stanford, California, United States of America; 2 Instituto de Patologia e Imunologia Molecular da Universidade do Porto (IPATIMUP), Porto, Portugal; 3 Department of Statistics, Stanford University, Stanford, California, United States of America; 4 HudsonAlpha Institute for Biotechnology, Huntsville, Alabama, United States of America; 5 Department of Health Research and Policy, Stanford University School of Medicine, Stanford, California, United States of America; 6 Centro de Investigação em Biodiversidade e Recursos Genéticos (CIBIO), Vairão, Portugal; 7 Universidade de Cabo Verde (Uni-CV), Praia, Santiago, Cabo Verde; 8 Gregor Mendel Institute, Austrian Academy of Sciences, Vienna, Austria; 9 Department of Anthropology, The Pennsylvania State University, University Park, Pennsylvania, United States of America; 10 Departamento de Biologia, Faculdade de Ciências da Universidade do Porto, Porto, Portugal; University of Colorado Denver, United States of America

## Abstract

Variation in human skin and eye color is substantial and especially apparent in admixed populations, yet the underlying genetic architecture is poorly understood because most genome-wide studies are based on individuals of European ancestry. We study pigmentary variation in 699 individuals from Cape Verde, where extensive West African/European admixture has given rise to a broad range in trait values and genomic ancestry proportions. We develop and apply a new approach for measuring eye color, and identify two major loci (*HERC2*[*OCA2*] *P* = 2.3×10^−62^, *SLC24A5 P* = 9.6×10^−9^) that account for both blue versus brown eye color and varying intensities of brown eye color. We identify four major loci (*SLC24A5 P* = 5.4×10^−27^, *TYR P* = 1.1×10^−9^, *APBA2*[OCA2] *P* = 1.5×10^−8^, *SLC45A2 P* = 6×10^−9^) for skin color that together account for 35% of the total variance, but the genetic component with the largest effect (∼44%) is average genomic ancestry. Our results suggest that adjacent cis-acting regulatory loci for *OCA2* explain the relationship between skin and eye color, and point to an underlying genetic architecture in which several genes of moderate effect act together with many genes of small effect to explain ∼70% of the estimated heritability.

## Author Summary

Differences in skin and eye color are some of the most obvious traits that underlie human diversity, yet most of our knowledge regarding the genetic basis for these traits is based on the limited range of variation represented by individuals of European ancestry. We have studied a unique population in Cape Verde, an archipelago located off the West African coast, in which extensive mixing between individuals of Portuguese and West African ancestry has given rise to a broad range of phenotypes and ancestral genome proportions. Our results help to explain how genes work together to control the full range of pigmentary phenotypic diversity, provide new insight into the evolution of these traits, and provide a model for understanding other types of quantitative variation in admixed populations.

## Introduction

Differences in human skin color have been shaped principally and dramatically by selection [1], [2], and therefore the underlying genetic architecture may inform disease-related traits such as obesity or hypertension, for which common susceptibility alleles may have been advantageous to early humans that came to inhabit different environments [3]. But our knowledge of skin and eye color genetics is incomplete, based mostly on the limited range of phenotypic diversity represented by individuals of European ancestry [4]–[10], or on candidate gene studies in African Americans [11]–[15]. Besides serving as a model system for gene action and genetic architecture, more knowledge about human pigmentary variation has the potential to provide important insights into human diversity, disentangling biology from both scientific and social stereotypes.

Cape Verde is an archipelago of ten islands located 300 miles off the coast of Western Africa. The previously uninhabited islands were discovered and colonized by the Portuguese in the 15^th^ Century, and subsequently prospered during the transatlantic slave trade. In this population, extensive genetic admixture between the African and European ancestral populations during the last several centuries [16], [17] has facilitated assortment of pigmentary alleles. Blond or red hair color is very rare in Cape Verde, but there is a wide spectrum of variation in both eye and skin color, and individuals with dark skin and blue eyes are not infrequent.

Pigmentary variation in Cape Verde also provides an opportunity to investigate and disentangle the effects of locus-specific vs. genome-wide ancestry effects, a problem that is especially relevant to admixed populations, which are poorly represented in existing GWAS efforts, and for whom health disparities often correlate with genome-wide admixture proportions. For example, in previous studies of African-American cohorts, darker skin color (used as a proxy for genome-wide ancestry) correlates with higher blood pressure and with lower socioeconomic status, but the extent to which genetic factors contribute to these correlations is unclear [18].

Previous studies of African-European skin color variation have focused mostly on candidate genes that exhibit large allele frequency differences between ancestral populations, and have led to the view that a small number of loci account for most of the phenotypic variation [13], [15], in which case skin color should correlate poorly with genome-wide ancestry. Availability of dense genotyping information and new analytical tools allow a more rigorous approach in which the effects of individual loci and genome-wide ancestry can be disentangled and comprehensively investigated.

In an admixed population, the effects of individual loci on a quantitative trait can be detected either by a correlation between genotype and phenotype, or by a correlation between local ancestry and phenotype. Genotype-based approaches are expected to be more powerful for traits where the causative allele exists at similar frequencies in ancestral populations, while ancestry-based approaches should be more powerful for traits where the causative allele exhibits large frequency differences in ancestral populations. Here we apply and compare genotype-based and ancestry-based association approaches for skin and eye color in 699 Cape Verdean individuals; both approaches identify two major loci for eye color, and four major loci for skin color. Surprisingly, the genetic component with the greatest effect on skin color is not a single locus but average genomic ancestry, which, together with these four major loci, accounts for most of the estimated heritable variation. Our results indicate that Cape Verdean pigmentary variation is the result of variation in a different set of genes from those determining variation within Europe, suggest that long-range regulatory effects help to explain the relationship between skin and eye color, and highlight the potential and the pitfalls of using allele distribution patterns and signatures of selection as indicators of phenotypic differences.

## Results

### Genetic admixture in Cape Verdeans

We first investigated the pattern and distribution of continental ancestry in 699 Cape Verdeans for whom detailed pigmentary phenotype and high density genotype information was available, the latter obtained with the Illumina 1 M platform as described below. Data from CEU and YRI HapMap individuals [19] was used to partition each Cape Verdean individual's ancestry into “European” and “African” components. We note that there is population substructure and pigmentary heterogeneity within Europe and, of course, within Africa. We also note that the true ancestral populations for Cape Verde are likely to include contributions from several areas of Southern Europe, and several areas of West Africa. In what follows, we use the terms “European” and “African” to refer to different continental origins, and emphasize that our use of these terms should not be taken to infer homogeneity of genotype or phenotype for the true ancestral populations of Cape Verde.

**Figure 1:**
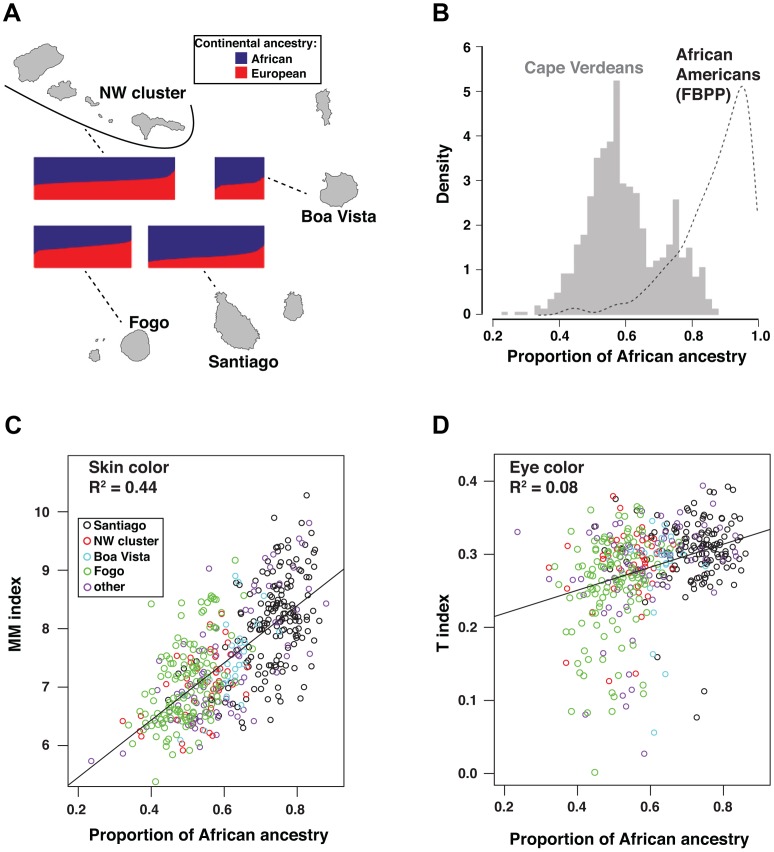
Relationship of geography and ancestry to skin and eye color. Individual ancestry proportions for Cape Verdeans displayed on all four panels were obtained from a supervised analysis in *frappe* with K = 2 and HapMap's CEU and YRI fixed as European and African parental populations. (a) Bar plots of individual ancestry proportions for Cape Verdeans across the islands. The width of the plots is proportional to sample size (Santiago, n = 172; Fogo, n = 129; NW cluster, n = 192; Boa Vista, n = 27). The proportion of African vs. European ancestry of the individuals is indicated by the proportion of blue vs. red color in each plot. (b) Individual African ancestry distribution in the total cohort of 685 Cape Verdeans (histogram) and in 802 African Americans (kernel density curve) from the Family Blood Pressure Program (FBPP) [21]. (c) Scatter-plot of skin color vs. Individual African ancestry proportions. Skin color is measured by the MM index described in Material and Methods. (d) Scatter-plot of eye color vs. Individual African ancestry proportions. Eye color is measured by the T-index, described in Figure 2 and Material and Methods. Points in scatter-plots are color coded according to the island of origin of the individuals.

The program *frappe* [20] implements a maximum likelihood clustering approach to infer individual genomic ancestry proportions. Overall, African genomic ancestry in Cape Verdeans ranges from 23.5% to 87.9%, with a median of 58%. Across different islands, the distributions of African genomic ancestry exhibit substantial overlap in range but vary in their median values, from 50.5% in Fogo to 74.4% in the capital island of Santiago (Figure 1a), which suggests a population history of extensive intercontinental admixture accompanied by reduced gene flow between islands.

The pattern of African genomic ancestry in Cape Verde is less skewed than in many other African-European populations. For example, in a randomly sampled African-American cohort from the Family Blood Pressure Study (FBPP) [21], African genomic ancestry ranges from 40.6% to 99.3%, with a median of 89.5% (Figure 1b).

The broad range of genomic ancestry makes the Cape Verde population particularly well suited for understanding the genetic basis of Afro-European phenotypic diversity; at the same time, careful adjustment for population stratification is critical in an association analysis. The genome-wide association results described below are based on a linear regression adjusting for the first three principal components (PC) but the same model adjusting for up to 10 PCs does not qualitatively alter our results. Additionally, we have taken two complementary approaches: (1) a linear regression adjusting for African genomic ancestry and self-reported island of birth, and (2) a mixed effect model that considers relatedness and stratification, implemented in the program, EMMAX [22]. All three approaches—genotype-based association, ancestry-based association, and a mixed model—point to the same set of genetic loci.

### Quantitative assessment of pigmentary phenotypes

For skin color, we used reflectance spectroscopy on the upper inner arm to calculate a modified melanin (MM) index, which appears normally distributed with a mean of 7.39 and standard deviation of 0.85 for the Cape Verde cohort. By comparison, MM in individuals of European ancestry is smaller and much more narrowly distributed, with a mean of 5.38 and standard deviation of 0.27 [23].

**Figure 2:**
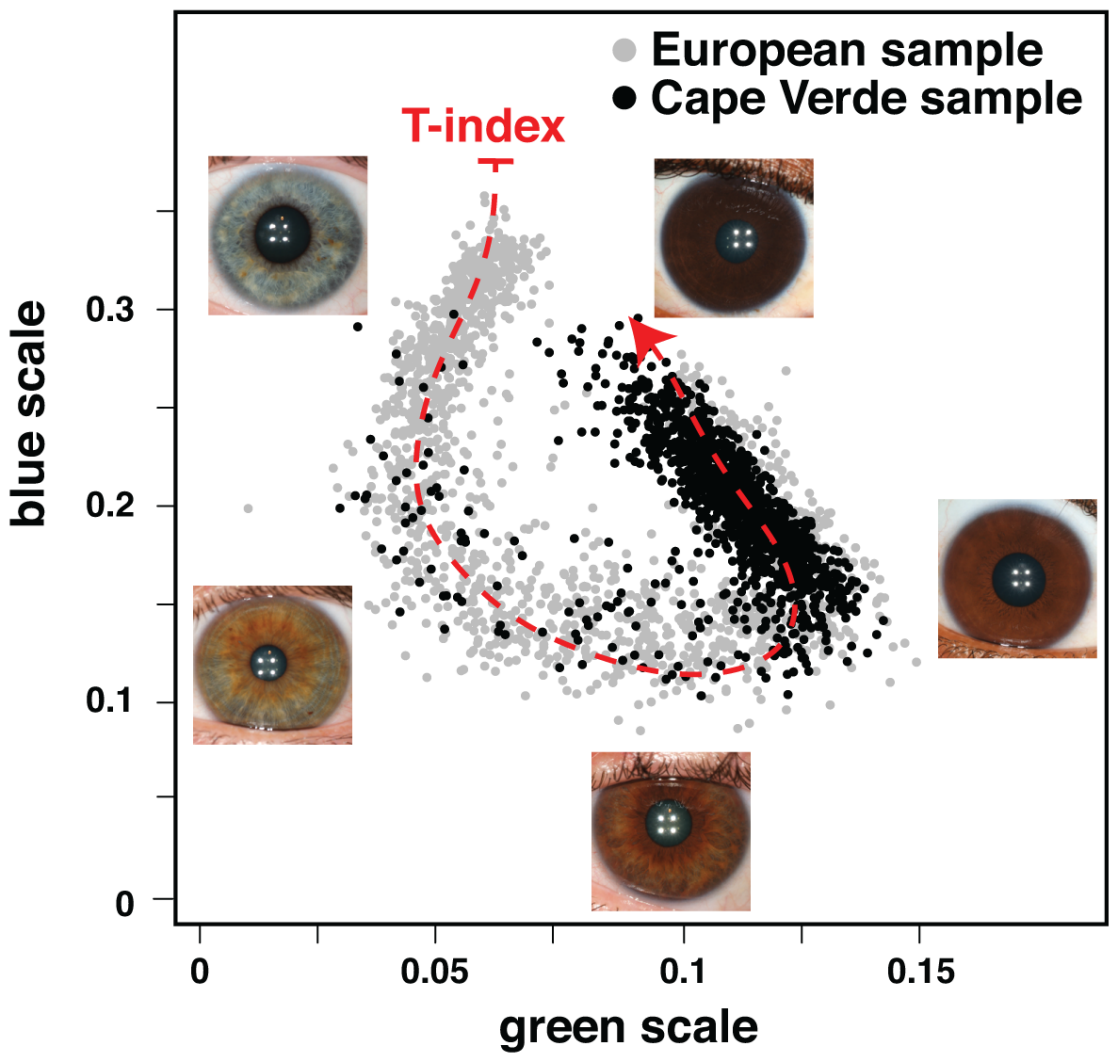
Quantitative assessment of eye color. Plotted are the normalized median values of green (x-axis) and blue (y-axis) levels of each individual's irises. We fitted a principal curve that explains most of the variation in the data (red dashed curve). The T-index is defined by the arc-length from the projection of each point on the curve to the end of the curve that corresponds to the lightest eye color. In the figure are examples of eye photos at their respective position in the T-index curve.

For eye color, we developed a new measure based on automated analysis of digital photographs that captures the full range of African-European variation. We observed that RGB reflectance values project onto an empirical curve that begins and ends, respectively, with very light blue, and very dark brown eyes (Figure 2). We describe the distance along this curve as the “T index”, a quantitative measure in which the categorical descriptions of “hazel”, “light brown”, and “medium brown” progressively increase in value (Figure 2).

The correlation between skin color and African genomic ancestry (R∧2 = 0.44) is apparent both across and within (Table S1) islands clusters, and is higher than anticipated for a trait determined by the action of a small number of genes. (For example, in a model with 3 major skin color genes that act additively and equally, we predict an R∧2<0.3 between African genomic ancestry and skin color [see Material and Methods]). The strong effect of genomic ancestry on skin color is also striking in the context of eye color; there is only a weak correlation between skin and eye color in Cape Verdeans (R∧2 = 0.14), and African genomic ancestry is also weakly correlated (R∧2 = 0.08) with eye color (Figure 1c, 1d). Overall, these observations point to different genetic architectures for skin and eye color.

### Mapping loci associated with skin and eye color

**Figure 3:**
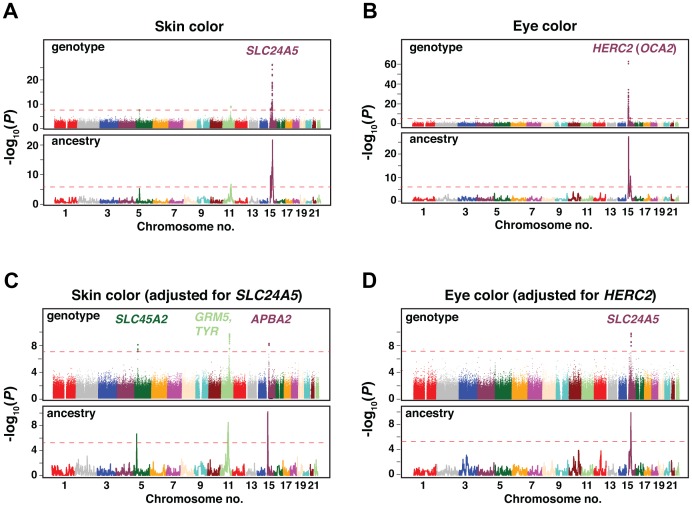
GWAS results for skin and eye color in the total Cape Verdean cohort. Results are shown as −log_10_(*P* value) for the genotyped SNPs. Plots are ordered by chromosomal position. (a,c) Genotype and admixture association scan results for skin color. (b,d) Genotype and admixture association scan results for eye color. (a,b) show the *P* values obtained in the initial scans and (c,d) the *P* values of the following scans adjusting for the strongest associated SNP (in *SLC24A5* for skin color and in *HERC2* for eye color). Dashed red lines correspond to the genome-wide significance threshold (P<5×10^−8^ in the genotype scan; *P*<7×10^−6^ in the ancestry scan [see Material and Methods]). The location and identity of candidate genes are colored to correspond with chromosomal location; individual SNPs are given in Table 1.

We used information from 903,837 autosomal SNPs to carry out genotype- and ancestry-based association analyses for skin (n = 685) and eye (n = 625) color. For genotype-based association, four skin color loci (5p13.3, 11q14.3, 15q13.1, and 15q21.1) and two eye color loci (15q13.1 and 15q21.1) met genome-wide criteria for significance, *P*<5.7×10^−8^ (Table 1, Table S2). In analyses adjusted only for principal components and sex, the signals at 15q21.1 (for skin color, Figure 3a) and 15q13.1 (for eye color, Figure 3b), dominate the significance plots. As described below, these signals correspond to *SLC24A5* for skin color and *HERC2* (*OCA2*) for eye color; Figure 3c and 3d show *P* value distributions when genotypes at *SLC24A5* and *HERC2* (*OCA2*) are considered as covariates. No additional loci were found in conditional analyses that adjust for the most significant SNPs in the known loci (four for skin and two for eye color). After removing SNPs surrounding these six loci, the genomic control parameters were 1.02 and 1.00 for skin color and eye color, respectively. We also examined the six loci identified in the combined sample in each island separately (Table S1); the direction of effect is consistent across all groups, with the derived allele associated with lighter skin color or a lower T-index (Table 1). Taken together with similar results from EMMAX (Figure S1), these results suggest adequate correction for population structure.

**Table 1:** Major loci for skin and eye color.

Chrom. region	Candidate gene(s)	SNP	Significance^a^		Phenotypic effect^b^	Derived allele freq.^c^		
			genotype	ancestry		EUR	AFR	CV
**Skin color**								
15q21.1	*SLC24A5*	rs1426654	5.4×10^−27^	3.9×10^−24^	−/−/−/−	1.00	0.01	0.50
11q14.3	*GRM5, TYR*	rs10831496^d^	1.1×10^−09^	1.2×10^−08^	−/−/−/−	0.66	0.10	0.37
15q13.1	*APBA2*	rs4424881^d^	1.5×10^−08^	6.1×10^−09^	−/−/−/−	0.85	0.05	0.41
5p13.3	*SLC45A2*	rs35395^d^^,^ f	6.0×10^−09^	5.3×10^−08^	−/−/−/−	0.99	0.25	0.69
**Eye color**								
15q13.1	*HERC2, OCA2*	rs12913832	2.3×10^−62^	2.3×10^−27^	−/−/−/−	0.79	0.00	0.18
15q21.1	*SLC24A5*	rs2470102^e^	4. 8×10^−10^	1.6×10^−10^	−/−/−/−	0.99	0.01	0.50

a *P* value for association of skin (n = 685) or eye (n = 625) color with genotype or local ancestry, adjusted for sex and the first three principal components, as described in Materials and Methods.

b Sign of the beta value in four groups (combined sample/Santiago/Fogo/Northern Islands). A “–” sign indicates that the derived allele causes lighter skin or a lower T index (Table S1).

c EUR—individuals with Northern and Western European ancestry, HapMap phase III CEU; AFR—individuals with West African ancestry, HapMap phase III YRI; CV—Cape Verdeans.

d *P* values obtained after controlling for *SLC24A5*, in addition to the other covariates. The corresponding values without controlling for *SLC24A5* are given in Table S2.

e *P* values obtained after controlling for *HERC2*, in addition to the other covariates. The corresponding values without controlling for *HERC2* are given in Table S2.

f After imputation, the most strongly associated SNP at 5p13.3 is the non-synonymous variant *SLC45A2* L374F (rs16891982), previously associated with pigmentation [27]. The frequencies of the derived, lightening, alleles are 0.98 and 0.00 in EUR and AFR, respectively.

For ancestry-based mapping, we used SABER+, an extension of a Markov-Hidden Markov Model method [24], to assign African vs. European ancestral segments along each chromosome, and then carried out a genome-wide evaluation of local ancestry association with skin and eye color, using the same covariates as for the genotype-based analysis. Ancestry-based association yields broader peaks than genotype-based association, but the distribution of significant regions is strikingly consistent for the two approaches, uncovering the identical set of six loci (Figure 3, Table 1).

### Refinement of pigmentation loci by imputation and conditional analysis

**Figure 4:**
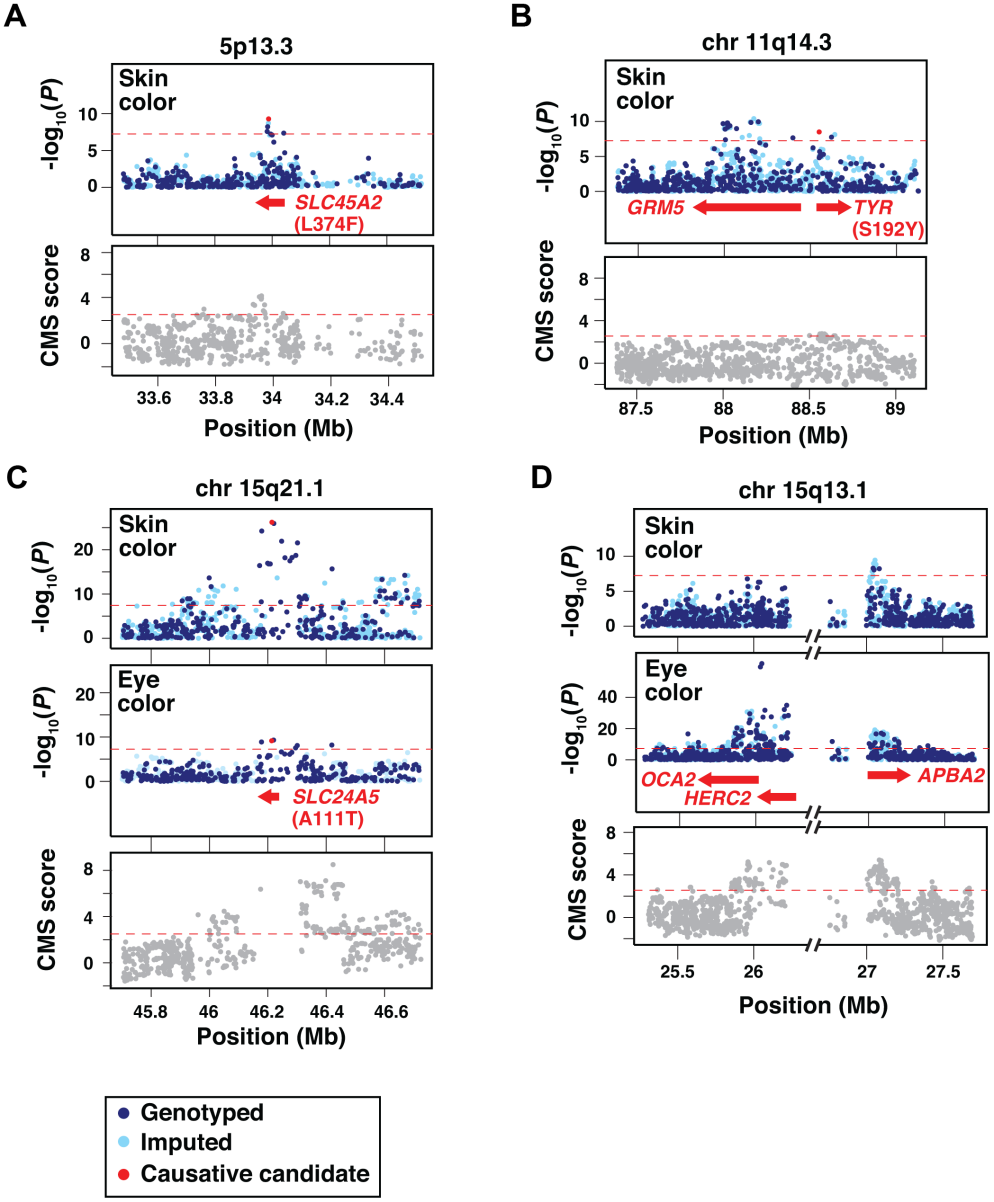
Imputation, fine-mapping, and selective signature scores for skin and eye color loci. Each panel shows the genotype-association results and the distribution of Composite of Multiple Signals scores of positive selection (CMS scores) [38] for genotyped and imputed SNPs (dark and light blue points in the association plots, respectively) surrounding the candidate loci; identities of individual SNPs are given in Table 1. Dashed red lines correspond to the genome-wide significance thresholds. (a) 5p13.3 region containing the *SLC45A2* gene. (b) 11q14.3 region containing the *GRM5* and *TYR* genes. (c) 15q21.1 region containing the *SLC24A5* gene. (d) 15q13.1 region containing the *OCA2*, *HERC2* and *APBA2* genes. The interval between *HERC2* and *APBA2* contains a cluster of segmental duplications and a paucity of SNPs [45], and is also a source of a frequent deletion breakpoint as described in the Discussion. Previously identified candidate causative non-synonymous variants [11], [13], [14] are denoted as red dots in the association plots of panels (a,b,c). The location and identity of candidate genes are colored to correspond with chromosomal location; individual SNPs are given in Table 1.

To help evaluate potential causative loci and molecular variants for skin and eye color, we imputed SNP information from HapMap phase II [19], repeated the genotype-based association, and considered the results in the context of existing knowledge and haplotype structure. The 5p13.3 and 11q14.3 regions affect only skin color (Figure 4a, 4b), whereas the chromosome 15 regions (15q13.1 and 15q21.1, Figure 4c, 4d) affect both skin and eye color. As described below, the 15q21.1 region harbors coding sequence variation in *SLC24A5* that affects skin and eye color similarly, but the 15q13.1 region harbors two distinct peaks of regulatory sequence variation that act separately on skin and eye color.

For skin color, the most strongly associated SNPs at 5p13.3 and 15q21.1 represent missense alterations in two well-known pigmentary genes, *SLC45A2* L374F (Figure 4a) and *SLC24A5* A111T (Figure 4c), thought to encode membrane transport proteins that promote melanogenesis [13], [25]–[27]. Neither amino acid substitution is predicted to dramatically alter protein function; nonetheless, expression of both genes is pigment cell-specific, and null mutations dramatically impair melanin synthesis in model organisms [13], [28], [29]. Thus, the European alleles probably represent hypomorphic alterations that compromise melanogenesis, leading to decreased pigmentation.

The two remaining skin color loci at 11q14.3 and 15q13.1 exhibit the strongest association signals within intronic regions of *GRM5* (Figure 4b) and *APBA2* (Figure 4d), neither of which is an obvious candidate to control normal variation in human skin color. However, *GRM5* and *APBA2* lie 396 kb upstream and 1025 kb upstream of *TYR* and *OCA2*, respectively, two well-known pigmentary genes for which a complete loss-of-function causes albinism in humans and in other animals [30]–[32]. Furthermore, a missense alteration in *TYR*, S192Y (rs1042602) [11] (Figure 4b), is in linkage disequilibrium with the most significant SNP in *GRM5* (rs10831496) in this sample, and in analyses that consider *TYR* and *GRM5* SNPs as covariates, both remain significant (*P* = 3.7×10^−6^ for rs10831496; *P* = 0.00012 for rs1042602). Thus, the effect of the 11q14.3 locus on skin color could be mediated by a combination of regulatory and coding variation.

The same is not true, however, for the 15q13.1 locus, for which the peak of skin color association in *APBA2* is separated from *OCA2* by ∼1 Mb (Figure 4d). This distinction is especially apparent when considering the 15q13.1 association signal for eye color, which lies ∼50 kb upstream of *OCA2* in an intronic region of *HERC2* (Figure 4d), and has been well recognized to account for Mendelian-like inheritance of blue vs. brown eye color in populations of European ancestry [7], [8], [10].

We considered whether the skin color GWAS signal for *APBA2* might simply reflect linkage disequilibrium with *HERC2* and *OCA2*, but rejected that hypothesis for several reasons. The GWAS significance peaks for eye color and skin color at 15q13.1 appear distinct (Figure 4d), and linkage disequilibrium between the two significance peaks is weak (R∧2 = 0.112 in our sample). Skin and eye color are, themselves, correlated, but in conditional regression analyses where genotypes at both loci are included as covariates, *HERC2* does not affect skin color if eye color is also included as a covariate (*HERC2 P* = 0.48, *APBA2 P* = 0.0009); conversely, *APBA2* does not affect eye color if skin color is also included as a covariate (*APBA2 P* = 0.083, *HERC2 P*<2×10^−16^). Thus, the effects of *HERC2* and *APBA2* are genetically and statistically separable. Still, there is strong experimental evidence that the *HERC2* region represents regulatory variation that acts via *OCA2* [33], and therefore we refer to these loci as *HERC2* (*OCA2*) and *APBA2* (*OCA2*) to indicate both their proximity to a well-known pigmentation gene, and a likely molecular mechanism.

In addition to the major eye color locus at *HERC2* (*OCA2*), there is a second eye color locus at 15q21.1 whose position corresponds to the *SLC24A5* A111T allele (Figure 4c). The association of *HERC2* and *SLC24A5* with eye color is also apparent in individuals who do not have blue or green eyes: In the subset of 592 Cape Verdeans whose T-index >0.15 (Figure 1, Figure 2), both loci remain highly significant (*HERC2* rs12913832, *P* = 5.23×10^−16^; and *SLC24A5* rs2470102, *P* = 1.12×10^−10^), indicating that variation at these loci affects different shades of brown eye color.

### Selection for pigmentary loci

Population genetic signatures of positive selection have been used as ancillary evidence to identify and/or refine pigmentation genes [34]–[37]. We examined how a recently described indicator of recent positive selection, the composite of multiple signal (CMS) scores, is distributed at each of the pigmentary loci identified by GWAS [38]. The CMS represents a combination of allele frequency and haplotype structure differences that arise when a variant that leads to increased fitness rapidly expands in the population, as is thought to have happened during the peopling of Europe for mutations that promote fair skin. We used genome-normalized CMS scores (personal comm., Grossman & Sabeti) from the HapMap CEU population; scores above 2.53 represent the upper 1% of the distribution, and are a sensitive indicator of loci that have undergone recent selection [38]. The *SLC45A2* and *APBA2* (*OCA2*) GWAS signals coincide with sharp CMS signals (Figure 4a, 4d); broader CMS signals are also apparent at *SLC24A5* and *HERC2* (*OCA2*) (Figure 4c, 4d). By contrast, very little evidence for positive selection is apparent in the *GRM5-TYR* region (Figure 4b). This difference—between pigmentary loci with and without signatures of selection— is not obviously related to effect size (for skin color, *SLC24A5* >> *SLC45A2*≈*GRM5-TYR* > *APBA2* (*OCA2*), see below), but, as expected, does correlate with African – European allelic divergence as estimated by HapMap YRI – CEU comparisons, for which the most highly associated SNP at each of the four skin color loci (Table 1) exhibits frequency differences of 0.99 (*SLC24A5*), 0.80 (*APBA2* [*OCA2*]), 0.74 (*SLC45A2*), and 0.56 (*GRM5-TYR*).

### Power to detect additional loci

**Figure 5:**
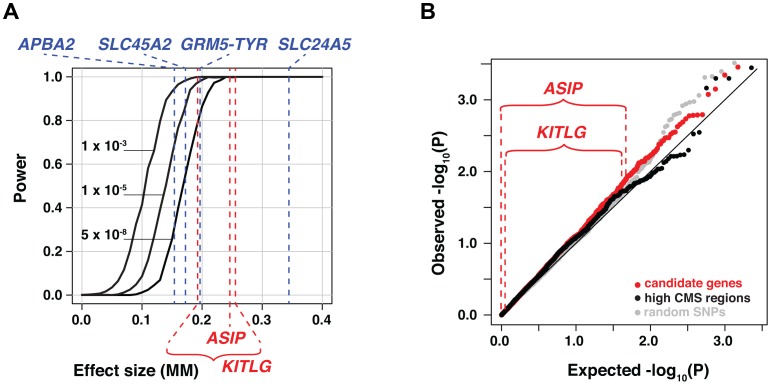
Power and candidate gene analyses. (a) Power estimated at three different alpha levels, plotted as a function of effect size (number of MM units by which each copy of the derived allele lightens skin pigmentation). The results shown here are based on derived allele frequencies of 0.1 and 0.9 in the ancestral African and European populations, respectively. The effect sizes detected in Cape Verde for the four major skin color loci are shown in blue; effect sizes for *ASIP* and *KITLG* as estimated (see Material and Methods) from [12] and [15], respectively are shown in red. (b) Distribution of *P* values for SNPs in 47 candidate genes, 16 regions with strong signatures of selection, and random SNPs, all shown as a q-q plot of the −log_10_ (*P*) values. Observed −log_10_ (*P*) values specifically for *ASIP* and *KITLG* SNPs are shown above the plot.

Notably absent from the four skin color loci detected in this study are *ASIP* and *KITLG*, reported previously to affect skin color in populations with African-European admixture [12], [15], and *IRF4*, *MC1R*, *SLC24A4*, *TYRP1*, reported previously to affect skin color in populations of European ancestry [4], [6]. To estimate the power of our study, we carried out simulations based on the observed distributions of individual genomic ancestry and genotypes for *SLC24A5*, in which the ability to detect a candidate locus at different levels of significance was evaluated as a function of effect size. For a genome-wide significance level (*P*<5×10^−8^), the results of our simulations reveal >90% power to detect an effect size of 0.21 MM (Figure 5a), which is 4.1% of the total range of skin color observed in our sample, and close to that suggested previously for *ASIP* (0.24 MM [12]) and *KITLG* (0.19–0.26 MM [15]).

We also asked if SNPs close to candidate genes might be enriched for low *P* values. In addition to *ASIP*, *IRF4*, *KITLG*, *MC1R*, *SLC24A4*, and *TYRP1*, we chose 41 additional candidate genes with a potential role in human skin color based on their phenotypes in model organisms, or in humans affected with albinism (Table S4). To evaluate candidate genes with no known role in pigmentation but which nonetheless have undergone recent selection, we used the XP-EHH metric to identify 16 regions harboring highly frequent and extended haplotypes in European compared to African populations (Table S5).

As shown in Figure 5b, the distribution of *P* values for SNPs in both types of candidate genes overlaps with that of randomly chosen genomic regions. As described further below, the failure to replicate previously reported effects could be due to a paucity of allelic variation in the Cape Verde ancestral populations (e.g. *MC1R*), incomplete correction for genomic ancestry with small panels of ancestry informative markers in the earlier studies (e.g. *ASIP*, *KITLG*), or real effects of these loci that are too small to detect in our study.

### Genetic and evolutionary relationships of eye and skin color genes at 15q13.1

As indicated above, one of the major skin color loci, *APBA2* (*OCA2*), lies close to but has effects that are genetically separable from, the major eye color locus, *HERC2* (*OCA2*). Selective pressure for fair skin in European ancestors is thought to reflect the need for sunlight-induced vitamin D production [1], [2], but there is no analogous hypothesis for selection of pale eye color; thus, a derivative *HERC2* (*OCA2*) allele may have hitchhiked on a derivative *APBA2*-bearing chromosome, or could have undergone independent selection for reasons that are distinct from those affecting *APBA2* (*OCA2*).

**Figure 6:**
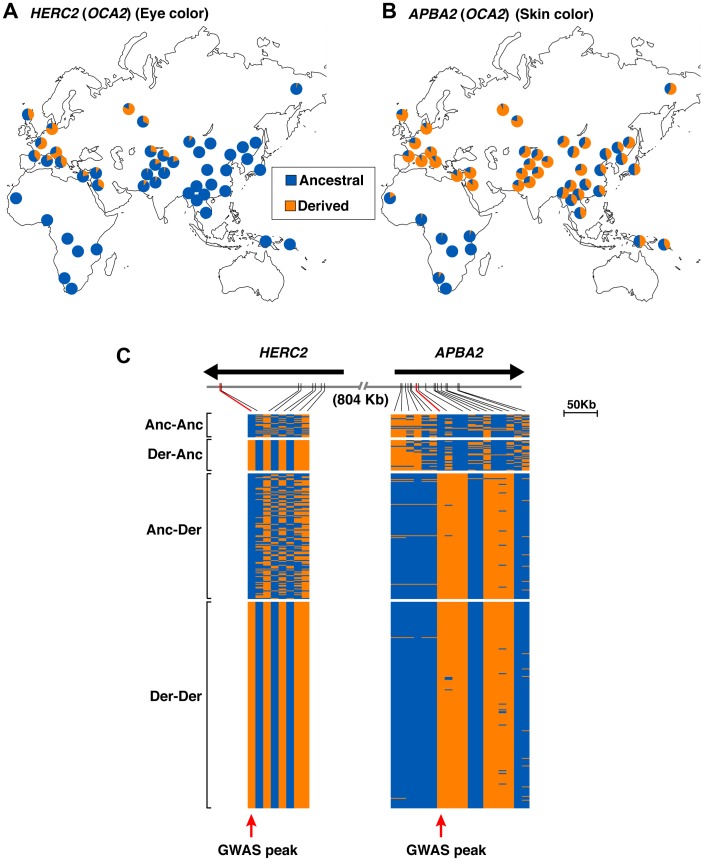
Allele frequency and haplotype analysis for eye and skin color loci at 15q13.1. (a,b) Allele frequency distributions for the SNPs most significantly associated with eye [*HERC2* rs1291382; (a)] and skin [*APBA2* rs4424881; (b)] in the HapmapIII [19] and HGDP [39] panels for the old world. Blue/orange shading corresponds to the frequency of the ancestral/derived alleles, as determined by comparison with the chimp reference sequence (assembly CGSC2.1/pan Tro2). Frequency values are presented in Table S3. c) Visual displays of the haplotypes extending from *HERC2* to the second intron of *APBA2* in Europeans from HapMap phase III (CEU) and HGDP (French, French Basque, North Italian, Tuscan, Sardinian, Orcadian, and Russian) panels. Haplotypes were inferred on the basis of 26 SNPs common to both datasets; blue and orange shades represent the ancestral and derived alleles, respectively. Haplotypes were ordered according to the ancestral/derived states at *HERC2* rs1291382 and *APBA2* rs4424881 (marked with red arrows), as follows: haplotypes bearing the ancestral alleles for both SNPs (Anc-Anc); haplotypes bearing the derived allele for *HERC2* rs1291382 and the ancestral allele for *APBA2* rs4424881 (Der-Anc); haplotypes bearing the ancestral allele for *HERC2* rs1291382 and the derived allele for *APBA2* rs4424881 (Anc-Der); and haplotypes bearing the derived allele for both SNPs (Der-Der).

To explore these ideas, we first examined worldwide allele frequency distributions for the most strongly associated SNP at each locus, using information from the Human Genome Diversity Project (HGDP) [39] and HapMap III [19]. The derived *APBA2* (*OCA2*) allele is present at low frequencies in most populations of African ancestry, and at high frequencies in most populations of Asian and European ancestry. By contrast, the derived *HERC2* (*OCA2*) allele is absent from African and East Asian populations, and appears at high frequency only in Western and Northern Europe (Figure 6a, 6b). These results suggest that an *APBA2* (*OCA2*) mutation conferring light skin arose before the spread of humans out of Africa, and that a *HERC2* (*OCA2*) mutation conferring pale eye color arose much later.

We then considered whether the light skin and pale eye color derived alleles were carried on the same haplotype in chromosomes of European ancestry, using information from HapMap CEU and European HGDP populations. Each derived allele is present on a single major extended haplotype (>160 kb in length for *HERC2* (*OCA2*) and >90 kb in length for *APBA2* [*OCA2*]), whereas the ancestral alleles are found on multiple, shorter, haplotypes. However, measures of linkage disequilibrium between the two loci are not significant (R∧2 = 0.036, D′ = 0.071 with Χ^2^ = 0.517), extended haplotypes for the derived alleles are separated by ∼800 kb, and approximately half of the European chromosomes we examined carry an ancestral *HERC2* (*OCA2*) haplotype together with a derivative *APBA2* (*OCA2*) haplotype, or vice versa (Figure 6c). These observations are consistent with the presence of discrete CMS peaks under the *HERC2* (*OCA2*) and *APBA2* (*OCA2*) regions (Figure 4d) and suggest that the loci may have undergone selection independently in European populations.

### Genetic architecture of pigmentary variation in Cape Verde

The four skin color loci we identified by association analysis act in an additive fashion: we found no evidence of dominance at any of the loci, nor evidence of opposite-sign epistasis between loci. For eye color, the ancestral *HERC2* (*OCA2*) allele is mostly dominant over the derived allele, consistent with the near recessive mode of inheritance of blue eye phenotype in Europeans (Figure S3). By contrast, the effects of *SLC24A5* on eye color are semi-dominant, and no interaction was found between this gene and *HERC2* (Figure S3).

Quantitative assessment of the skin color loci can be considered from two perspectives: effect size in an individual, and contribution to total phenotypic variance in a population. The former depends only on genotype, and measures the strength of allelic substitution, whereas the latter is influenced by the distributions and potential correlations of allele frequencies in the specific population being studied. From the first perspective, alleles of the four major loci shift the MM index 0.16–0.33 units (in Cape Verde, skin color ranges from ∼5.5–10.5 units), and the sum of effect sizes for homozygous substitution at all four loci would shift, in an individual, an amount about the same as ∼1/3 of the total range of skin color we observed (Figure 6a).

From the second perspective, the proportions of phenotypic variance attributed solely to genotype at each of the four major skin color loci are quite small, about 2% each for *GRM5-TYR*, *APBA2* (*OCA2*), and *SLC45A2*, and about 7% for *SLC24A5*. However, these estimates, based on conventional regression analyses in which individual genomic ancestry is considered as a covariate, fail to consider admixture stratification, wherein admixture proportions vary widely due to recent admixing, and genotypes at unlinked loci remain correlated. For example, even though *SLC24A5* and *SLC45A2* lie on different chromosomes, their genotypes in the Cape Verde population are correlated with genomic ancestry and, therefore, with each other. Thus, in any particular individual, genomic ancestry has predictive value for genotype at each of the four major skin color loci, and vice versa.

**Figure 7:**
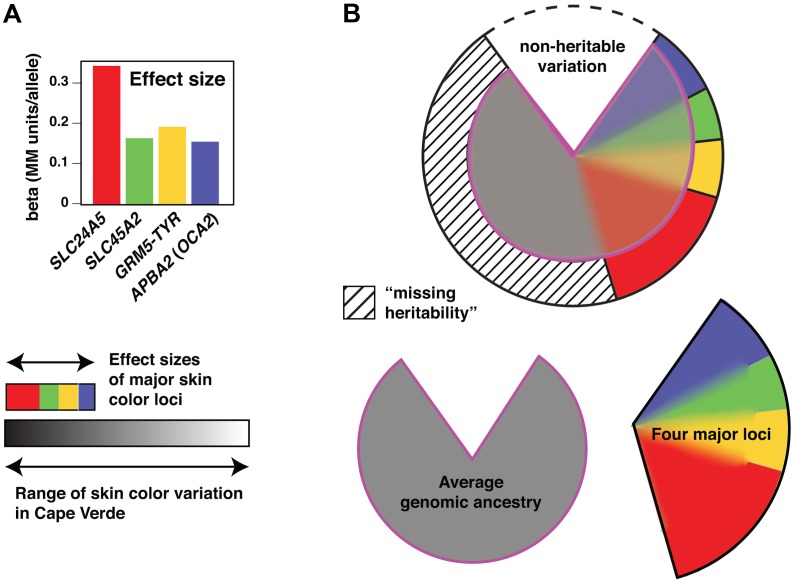
Genetic architecture of skin color variation. (a) Effect sizes of the loci associated with skin color. Effect values represent the beta values obtained from a regression model containing the four associated loci plus ancestry. (b) The pie chart represents the proportion of phenotypic variation accounted for by the different components, including non-heritable factors (∼20%), the four major loci (∼35%, color-coded as in [a]), and average genomic ancestry (44%). The heritable contributions were estimated by regression and variance decomposition as described in Material and Methods, and are also represented below the pie chart separately as grey (genomic ancestry) or open (four major loci) areas. However, because of admixture stratification, the heritable contributions overlap as described in the text.

To capture this predictive value, we applied the epidemiological concept of population-attributable risk—the extent that an environmental risk factor contributes to phenotypic variance in a population—to determine the quantitative impact of allelic substitution at each of the four major loci. This quantity, to which we refer as “population-attributable variance”, is calculated by determining the fractional reduction in phenotypic variance that would occur if genotypes at a locus of interest were set to a common baseline in all individuals. For skin color, population-attributable variances of *SLC24A5*, *GRM5-TYR*, *APBA2* (*OCA2*), and *SLC45A2* are 18%, 7%, 9% and 7%, respectively, considerably less than the proportion of variance attributed to individual genomic ancestry, 44% (Figure 7b, also Figure 1c).

These quantitative genetic relationships and their contributions to skin color variation in Cape Verde are depicted in Figure 6 as overlapping regions; the four major loci contribute a total of 35% to skin color variation, but only 13% above that which could also be accounted for by individual ancestry. Conversely, even though individual ancestry contributes 44% to skin color variation, about half of that contribution (22% of the total) could also be accounted for by genotype at the four major loci.

The aforementioned discussion considers the different components as a proportion of total variation, not all of which is heritable. Previous studies of skin color estimate narrow sense heritability at 70%–90% of total variation [40], and genotype of the four major loci together with individual ancestry accounts for 57% of total variation. If skin color in Cape Verde is 80% heritable, the four major loci and individual ancestry explain most of that variation, with only a small amount of “missing heritability” (the hashed portion of Figure 7b).

## Discussion

Previous genome-wide studies of human skin and eye color have focused on populations of European ancestry and have been based primarily on categorical and subjective assessment of pigmentary phenotype [4]–[7]. The work reported here—in which the choice of population, phenotype assessment, and statistical analysis strategies were developed to provide robust information across a broad range of genetic and phenotypic backgrounds—reveals a likely new regulatory locus and mechanism of action, provides a more refined view of the underlying genetic architecture than previously appreciated, and has important implications for studying quantitative phenotypes in admixed populations.

*OCA2* was originally considered a strong candidate gene for Afro-European pigmentary variation based on allele distribution patterns of closely linked SNPs [11], [14], [34], [35], and because of a strong association with blue vs. brown eye color in populations of European ancestry [4], [41]. This eye color locus was later refined to a small region 50 kb upstream of *OCA2* in an intron of the *HERC2* gene [7], [8], [10]. Very recently, Visser et al. [33] showed that the critical SNP in this region, rs12913832, is part of an enhancer that forms a chromatin loop with the *OCA2* promoter, and that the derivative allele causes reduced recruitment of the chromatin remodeler HLTF, leading to reduced binding of additional transcription factors and impairment of loop formation and *OCA2* expression in cultured human melanocytes.

Our work confirms the very strong effect of rs12913832 on eye color but provides little evidence for an effect on skin color, since neither the transcribed region of *OCA2* nor *HERC2* has a major influence on skin color in Cape Verde. Instead, the 15q13.1 skin color locus maps to an intronic region of *APBA2* that lies *∼*1 Mb away from *OCA2*, and has a haplotype set, linkage disequilibrium architecture, and signature of selection that is clearly distinct from the *HERC2* (*OCA2*) eye color locus. Neither functional nor expression studies suggest a role for the APBA2 protein in pigmentation [42]–[44]; instead, we favor the hypothesis that the action of *ABPA2* (*OCA2*) is analogous to that of *HERC2* (*OCA2*), with distinct cis-acting regulatory regions that can affect eye and skin color independently. Critical evaluation of this hypothesis will require functional studies similar to those described by Visser et al. [33], but it is interesting to note that the ∼1 Mb interval between *HERC2* and *APBA2* contains a cluster of segmental duplications that serves as a frequent deletion breakpoint (BP3) involved in Prader-Willi and Angelman syndromes [45]. Hypopigmentation associated with these conditions is thought to be caused by hemizygosity for *OCA2* [46] and is therefore independent of any potential cis-acting regulatory region in *APBA2*. If, however, a chromatin loop does form between *APBA2* and *OCA2*, the BP3 region must be contained within that loop.

Furthermore, the notion that expression of *OCA2* can be modulated and selected separately in specific pigment cell compartments could help to explain the correlation and evolutionary history of skin and eye color. In particular, loss-of-function for *OCA2* can cause blindness due to loss of melanin from retinal pigment epithelial cells [31], and we speculate that natural selection for the *APBA2* region in Europeans represents cell type-specific regulation that promotes fair skin while preserving visual function. Finally, our population genetic analysis of *HERC2* and *APBA2*, together with similar work on the *HERC2* - *OCA2* interval from Donnelly et al. [37], is consistent with the possibility that blue eyes were selected independently from fair skin for reasons having to do with reproductive success rather than survival.

Our analysis of skin color genetic architecture generally assigns smaller contributions to individual genes than in previous work. In particular, *SLC24A5* and *KITLG* have been reported to have effect sizes that correspond to 25%–38% and 20%–25% of the European-West African difference in skin color, respectively [13], [15]. In our work, the effect size of *SLC24A5* corresponds to 13% of the range of skin color variation in Cape Verde, and we did not detect *KITLG* as a skin color locus. For *SLC24A5*, this apparent discrepancy stems from whether effect sizes are described as a fraction of the “average pigmentation difference between European-Americans and African Americans of about 30 melanin units” [13], or the entire dynamic range within a population, which is 77 melanin units in Cape Verde. Indeed, we estimate an effect size for a single *SLC24A5* allele in Cape Verde that corresponds to 4.9 melanin units, compared to 4.8 melanin units estimated by Lamason et al. [13] in an African-American sample. This somewhat trivial explanation underscores the importance of how quantitative genetic effects are described, reported, and interpreted, especially when the results have potential explanatory and predictive value for morphologic traits that distinguish different biogeographic ancestries.

Admixture stratification is important in this context because the very same loci that underlie trait differences in ancestral populations are also among the strongest admixture informative markers; therefore, incomplete correction for genomic ancestry will lead to overestimation of the effects for any locus that itself is an admixture informative marker. A potential example is apparent from *KITLG*. Like *APBA2* (*OCA2*), *SLC24A5*, and *SLC45A2*, SNPs in *KITLG* exhibit large CEU-YRI allele frequency differences of 0.7–0.99. In the Cape Verde sample, we used dense genotype data (>900,000 SNPs) to correct for genomic ancestry, and only the first three loci exhibited a significant association with skin color. By contrast, the observations of Miller et al. on the effects of *KITLG* in an African-American sample used a relatively small set of admixture informative markers to correct for genomic ancestry, which may have led to an overestimation of effect size. Our results do not exclude the possibility that variation in *KITLG* affects skin color—indeed, like other genes in which mutations cause strong pigmentary effects in model organisms (Table S4), *KITLG* remains a good candidate gene; however, any effect in the Cape Verde population is too small to be detected in our sample.

Although eye color and skin color are correlated, their underlying genetic architecture in Cape Verde is very different. Beyond *HERC2* (*OCA2*) and *SLC24A5*, individual genomic ancestry has relatively little effect on eye color. By contrast, the “rest of the genome” (beyond *APBA2* (*OCA2*), *GRM5-TYR*, and *SLC24A5*) has a very strong influence on skin color, nearly twice that of all four single loci combined. What is the biological basis for the large effect of individual ancestry on skin color? Theoretical explanations include non-genetic components that correlate with ancestry, or many genes of small effect whose contributions were not strong enough to be detected in our sample. We favor the latter alternative, which supports a view of skin color genetic architecture that is multimodal, in which many genes of small effect cumulatively explain the correlation between skin color and individual ancestry, and together with several genes of moderate effect (*GRM5-TYR*, *APBA2* (*OCA2*), *SLC45A2*, and *SLC24A5*), account for high overall heritability. These observations also have important implications for potential forensic applications, and argue that efforts to predict pigmentary phenotype from genotype should be based on dense genotype and/or whole genome sequence information rather than small panels of SNPs.

Human history has given rise to group-level differences in both phenotype and genotype, and recently admixed populations such as Cape Verde offer the potential to unravel the causal relationships. But relying primarily on differences in population-based allele frequencies or haplotype architecture to infer causes of morphologic variation is a form of genetic stereotyping that can lead to erroneous conclusions. An accurate understanding of human diversity requires that we measure phenotype as well as genotype.

## Materials and Methods

### Study population and genotyping QC

Cape Verde individuals were recruited from six islands of the archipelago (Figure 1a). Each participant reports age, sex and place of birth. DNA was extracted from finger-stick blood collected and stored on FTA paper, with the FTA Purification Reagent (Whatman Inc., Piscataway, NJ) and according to the manufacturer's protocol. Written informed consent was obtained from all participants and the study was approved by the Human Subjects Committees of all participant institutions and by the National Ethical Committee for Health Research of Cape Verde.

Genotyping was performed using the Illumina Infinium HD Human1M-Duo Beadarray (Illumina, San Diego, CA), according to the manufacturer's recommendations. A total of 1,016,423 SNP markers were assayed. SNPs with genotyping call rate <95%, SNPs showing replication errors across 20 replicated samples, SNPs in which the apparent heterozygosity was equal to one, and SNPs for which only a single allele was called in the Cape Verde dataset were removed from the analyses. Hardy-Weinberg equilibrium was not used as a quality filter due to the admixed nature of the subjects. After applying all quality control filters, 903,837 autosomal SNPs were available for analysis, of which 24,478 had minor allele frequency (MAF) <0.01.

Two samples with call rate below 95% were removed. Identity-by-states (IBS), estimated using all autosomal SNPs in PLINK [47], revealed 12 pairs of cryptic relationships (IBS>0.8). One individual from each pair was excluded. The final dataset consists of 685 individuals with skin reflectance measurements, of which 625 have high-quality digital eye photographs.

### Phenotypes

Quantitative measures of skin pigmentation were taken on the upper inner arms using a handheld spectrophotometer (DSMII ColorMeter, Cortex Technology, Denmark). The instrument reports a melanin index (M index), which equals to 100× log (1/% melanin reflectance at 650 nM). For each subject, three consecutive measurements were taken on each arm. We calculated the mean of the six measurements using the Huber M-estimator (which is more robust against outlier measurements [48]); we report the square root of the mean as the MM index (which is more normally distributed than untransformed data).

For eye color, we developed an automated algorithm to compute quantitative measures of eye color based on the Red, Green, and Blue (RGB) values of full-eye digital photographs, taken at a fixed exposure setting. Briefly, we used a common landmark in the pupil to identify an area that corresponds to the left eye. We next delineated the inner and outer boundaries of the iris by seeking circular edges that mark sharp changes in RGB values. The extracted irises were manually inspected and found sufficiently accurate. The median values of the red, blue and green channels, over all pixels in the reconstructed iris region in an individual, were normalized to sum to one. We processed data from 1272 Cape Verdeans (of whom 625 were genotyped) with this approach.

We supplemented this dataset with manually curated RGB data from 1278 Europeans (including approximately equal numbers of individuals from Ireland, Italy, Poland, and Portugal [23]), 14 Africans, 19 East Asians, 10 Middle Easterners, 9 Indians, 1 Polynesian, 83 African-European admixed individuals, 10 East Asian-European admixed individuals, 9 European-Native American admixed individuals, 42 Hispanics, and 1 Indian-African admixed individual. A principal curve is then fitted to the green vs. blue scatter plot (Figure 2); this curve is chosen to be smooth and to capture maximum variation among all points. Each point, representing the median RGB value of a digital image, is projected to the principal curve. The T-index is defined by the arc-length of the point from the end of the curve that corresponds to the lightest eye colors. Ranges of 0–0.15 and 0.15–0.4 correspond roughly to blue/green and brown eye color, respectively (Figure 2).

### Characterization of genetic admixture

Individual genomic ancestry proportions for Cape Verdean individuals were estimated using program *frappe* [20], assuming two ancestral populations. HapMap genotype data, including 60 unrelated European-Americans (CEU) and 60 unrelated West Africans (YRI), were incorporated in the analysis as reference panels (phase 2, release 22) [19].

Although CEU and YRI are approximations of the true ancestral populations of Cape Verde, in previous work on admixed populations from Mexico [24], we have found that accurate local ancestry estimates can be obtained using imperfect ancestral populations (including CEU and YRI), as long as the haplotype phasing is accurate. We also note that genome-wide ancestry proportions estimated using CEU and YRI in *frappe* are highly correlated (r>0.988) with the first principal component computed on Cape Verdean genotypes alone without using any ancestral individuals. Thus, while the CEU and YRI are imperfect ancestral populations, they do not cause a large bias in either genome-wide or local ancestry estimates.

Locus-specific ancestry was estimated with SABER+, using the haplotypes from the HapMap project to approximate the ancestral populations. SABER+ extends a previously described approach, SABER, by implementing a new Autoregressive Hidden Markov Model (ARHMM), in which the haplotype structure within each ancestral population is adaptively learned through constructing a binary decision tree [24]. In simulation studies, the ARHMM achieves comparable accuracy as HapMix [49], but is more flexible and does not require information about the recombination rate. Both the *frappe* and SABER+ analyses included 537,895 SNP markers that are in common between the Cape Verdean and the HapMap samples.

Principal Component analysis (PCA) was performed using EIGENSTRAT [50]. Twelve individuals were removed because of close relationships (IBS>0.8). The first PC is highly correlated with African genomic ancestry estimated using *frappe* (r = 0.99).

### Association and admixture mapping

Association between each SNP and a phenotype (MM index for skin and T index for eye pigmentation) was assessed using an additive model, coding genotypes as 0, 1, and 2. Sex was adjusted as a covariate; age was found not correlated with the phenotypes (P>0.5 for both skin and eye colors), and hence was not included as covariate. Assessment and control for population stratification is described in Results; the *P* values reported in Table 1 and are derived from linear regressions using PLINK in which the first 3 principle components and sex are included as covariates. We also carried out an association analysis with the program EMMAX [22], which adjusts for population stratification by including a relationship matrix as a random effect; the results (Figure S1) were similar to those obtained using conventional association analysis (Figure 3).

We restricted the association scans to the 879,359 autosomal SNPs with MAF>0.01; SNPs achieving a P<5.7×10^−8^ were considered genome-wide significant. Conditional analyses were performed using a linear model that included the genotype at a major locus: *SLC24A5* for skin and *HERC2* (*OCA2*) for eye. To evaluate potential secondary signals, we also carried out an association scan conditioning at all index SNPs, and found no evidence for secondary signals except in the *GRM5-TYR* region (rs10831496 and rs1042602, respectively) as described in the conditional analysis section of the Results.

For ancestry mapping, which seeks statistical association between locus-specific ancestry and a phenotype, we used a linear regression model similar to that used in the genotype-based association, except substituting genotype with the posterior estimates of ancestry at a SNP, estimated using SABER+; again, sex and the first three PCs were used as covariates. Based on a combination of simulation and theory, we have previously established a genome-wide significant criterion of p<7×10^−6^ for this ancestry-based mapping approach [51].

### Power calculations and estimates of effect size

Simulated datasets were based on the observed distributions of genome-wide ancestry, *SLC24A5* genotypes, and skin color phenotypes. Specifically, local ancestry was first simulated from the known distribution of genome-wide ancestry, and the genotype at a candidate locus was then simulated using local ancestry and the estimated ancestral allele frequencies (based on CEU and YRI allele frequencies). Phenotype for each individual was then calculated from a linear model in which genome-wide ancestry, genotype at *SLC24A5* rs1426654, and genotype at the candidate locus were used as covariates together with a random error term whose variance was chosen so that the phenotypic variance of the simulated dataset matched the variance actually observed in the Cape Verde sample. This approach preserves a realistic level of correlation structure between phenotype, genome-wide ancestry proportions and genotypes, and also takes into account the two strongest predictors of phenotype: genome-wide ancestry and genotype at SLC24A5. The linear model for calculating phenotype used regression coefficients of −4.247 for genome-wide European ancestry and −0.3459 per copy of *SLC24A5* rs1426654 derived allele; for the candidate locus, we varied the regression coefficient to evaluate power for different effect sizes.

A linear regression model was then used to test the association between phenotype and genotype, and power calculated as the proportion of 1000 simulations in which the association exceeded a pre-specified alpha-level. Effect sizes shown in Figure 5 and Figure 7 for *APBA2* (0.155) *GRM5-TYR* (0.193), *SLC24A5* (0.334), and *SLC45A2* (0.166) are from a linear regression of the Cape Verde skin color data in which genomic ancestry and genotypes at the four major loci are included as covariates. Effect sizes shown in Figure 5 for *KITLG* (0.19 MM–0.26 MM) are estimated from Miller et al. [15] in which the beta value for rs642742 is reported as 3.8 melanin units based on a linear regression model, and 2.8 melanin units based on ADMIXMAP. Similarly, the effect size shown in Figure 5 for *ASIP* (0.24 MM) is estimated from 3.5 melanin units based on data in Bonilla et al. [12].

The power values shown in Figure 5 are based on a single candidate locus with derived allele frequencies of 0.1 and 0.9 in the ancestral African and European populations, respectively, and are nearly identical to values based on candidate loci with the specific ancestral allele frequencies shown in Table 1, and *KITLG* allele frequencies of 0.056 and 0.827.

### Imputation, selection signature, and haplotype analysis

Imputation was performed for an ∼1–2.5 Mb window chosen to extend +/−500 kb of all genes identified in the association study. Imputed genotypes were inferred with the MaCH 1.0.16 package [52] using combined CEU and YRI haplotypes as reference; we report SNPs whose predicted R∧2 between true and imputed genotypes >0.80.

We directly compared association results for imputed genotypes with the composite of multiple signal (CMS) scores for positive selection developed by Sabeti and colleagues [38]. The results depicted in Figure 4 are based on genome-wide CMS scores kindly provided by S. Grossman and P. Sabeti for the HapMap CEU sample; this dataset exhibits a mean of 0 and variance of 1, and a CMS score of 2.53 corresponds to an empirical quantile of 99%.

Haplotype analysis of the 15q13.1 loci in Europeans was based on HapMap [19] CEU (phase 3) data supplemented with information from European HGDP populations (French, French Basque, North Italian, Tuscan, Sardinian, Orcadian, and Russian) for which haplotype structure was inferred with BEAGLE [53].

### Genetic architecture

Population-attributable variance refers to the proportion of phenotypic variation that can be accounted for by genotype of one or more loci, and is analogous to the epidemiologic concept of population-attributable risk. In the presence of admixture stratification, population-attributable variance for multiple loci sums to more than unity, because genotypes at unlinked loci are correlated with each other and with genome-wide ancestry.

We estimated population-attributable variance for the Cape Verde population by determining the fractional reduction in phenotypic variance that would occur if genotypes at a locus of interest were set to a common baseline in all individuals, holding all other covariates and genotypes unchanged. Specifically, for locus k and β_k_ (where β_k_ is estimated from a regression that includes all covariates and major locus genotypes), the fractional reduction in phenotype Y_[−k]_ = Y−β_k_G_k_, where Y is the observed phenotype, and G_k_ ε (0, 1, 2) is the genotype, calculated for each individual in the population. Population-attributable variance is then computed as PAV_k_ = [var(Y)−var(Y_[−k]_)]/var(Y). Analogously, the population-attributable variance for a set of loci is determined by setting the genotype of each locus to a common baseline simultaneously. Since population-attributable variance is computed only on variances of Y_[−k]_, the choice of the baseline genotype is irrelevant.

To estimate the proportion of phenotypic variation for a single locus that cannot otherwise be accounted for by genotype at unlinked loci, we compare the residual variance in a full regression model (that includes genotype at all loci and all covariates) to residual variance in a reduced model that does not include the locus of interest. Finally, variance accounted for by genome-wide ancestry is simply the R-squared statistic from a regression model that includes ancestry. We note that the PAV is non-additive; in other words, variance accounted by two loci is not the sum of the PAV of the two loci individually. To see this, consider a simpler situation with just two loci, with genotype G_1_ and G_2_, and let the true phenotype, Y, be 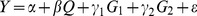.

The numerator of PAV for locus 1 is
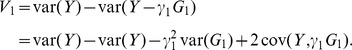



Similarly, we have for locus 2,
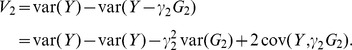



On the other hand, the numerator of PAV for loci 1 and 2 together is
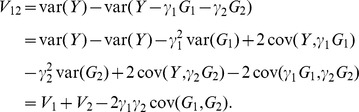



In our situation, if we code G_1_ and G_2_ to represent the derived (pigmentation lightening) alleles at the two loci, then cov(G_1_, G_2_)>0 due to admixture linkage disequilibrium, γ_1_γ_2_>0, and we have V_12_<(V_1_+V_2_), as is indicated by the overlapping areas in Figure 7.

To support the statement that correlation between skin color and African genomic ancestry (R^2^ = 0.44) is much higher than expected for a trait determined by the action of a small number of genes, we performed simulations using the observed genotype and ancestry information in the Cape Verde individuals. The phenotypes were simulated assuming a genetic architecture with three major loci, each of which has the same effect as *SLC24A5* and is perfectly ancestrally informative. The residual non-genetic variance was assumed to be independent of any genotype or ancestry, such that the total variance in each simulated phenotype matches that observed for skin pigmentation in Cape Verde. In 1000 simulations, the correlation between genome-wide ancestry and the simulated phenotype has a mean of 0.27 with a standard deviation of 0.037.

To exclude the possibility that the association between skin color and genome-wide ancestry is due to geographic confounding between islands, we examined this correlation within each island (Table S1).

We also considered using a mixed effects model, such as that implemented in program GCTA, to estimate genetic contributions to additive phenotypic variance as carried out by Visscher and colleagues [54]. However, the genetic relationship matrix based on allele sharing is inflated by population structure [55]; in addition, in pilot studies we observed a large sampling variability of GCTA-estimated heritability due to the moderate size of the Cape Verde sample.

### Evaluation of candidate loci

For pigmentary candidate loci, we chose 47 autosomal genes with a potential role in human skin color based on their phenotypes in model organisms, or in humans affected with albinism. For candidate loci based on signatures of selection, we used the XP-EHH metric to identify 16 regions harboring highly frequent and extended haplotypes in European compared to African populations. The lists of genes and their references are in Tables S3 and S4. For each candidate gene, we identified the physical position using NCBI36/hg18 assembly of the public genome reference sequence, extracted all the genotyped SNPs in the Cape Verdean cohort that fell within the genetic regions ±50 Kb to include putative regulatory regions, and carried out a genotype-association scan conditioning on the four major loci. We compared the obtained *P* value distributions for the candidate loci with the *P* value distribution obtained for a random set of SNPs spaced 1 Mb apart. The q-q plots for the three sets of SNPs are shown in Figure 5b, and allele frequency distributions are shown in Figure S2.

## Supporting Information

Figure S1 EMMAX results for skin and eye color in the total Cape Verdean cohort. Results are shown as −log_10_(*P* value) for the genotyped SNPs. Plots are ordered by chromosomal position. (a) Genotype association scan results for skin color. (b) Genotype association scan results for eye color. Dashed red lines correspond to the genome-wide significance threshold.
(TIF)

Figure S2 Distribution of allele frequencies for the two sets of candidate gene P values, and a control set of random SNPs (as depicted in Figure 5b). MAF, minor allele frequency. The distribution of the SNPs from the phenotype-based candidate genes is significantly different from the other two sets (*P* values for Kolmogorov-Smirnov tests are 0.01538 and 0.00944 for the comparison to selection-based candidate genes and random SNPs, respectively). The distribution of the SNPs from the selection-based candidate genes is not significantly different from the random SNPs (*P* = 0.125).
(TIF)

Figure S3 Effect of HERC2 (OCA2) and SLC24A5 genotype on eye color. Quantitative assessment of eye color with the T-index as described in the text and Figure 2.
(TIF)

Table S1 GWAS results and derived allele frequencies stratified by island cluster.
(DOCX)

Table S2 Major loci for skin and eye color (without conditioning).
(DOCX)

Table S3 Frequency of the derived allele for the eye and skin color loci at 15q13 in the Old World populations of HGDP and HapMap phase III datasets.
(DOCX)

Table S4 Candidate loci chosen based on function.
(DOCX)

Table S5 Candidate loci chosen based on selective signatures.
(DOCX)
