# Supplementary figures and images for: Genetic Architecture of Skin and Eye Color in an African-European Admixed Population
Source: PLoS Genet. 2013 Mar 21;9(3):e1003372. doi: 10.1371/journal.pgen.1003372 (PMC3605137; doi:10.1371/journal.pgen.1003372)

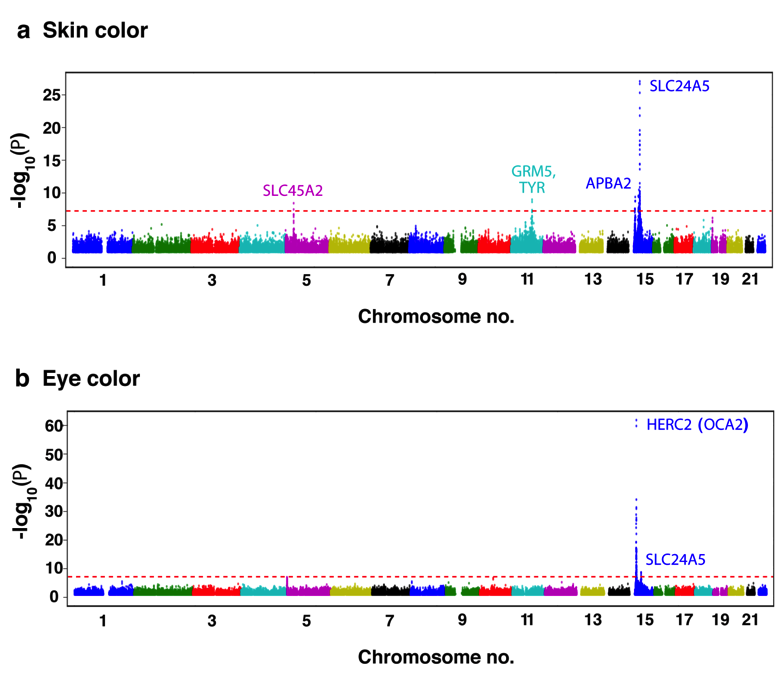

Supplement: Figure S1 — EMMAX results for skin and eye color in the total Cape Verdean cohort. Results are shown as −log10(P value) for the genotyped SNPs. Plots are ordered by chromosomal position. (a) Genotype association scan results for skin color. (b) Genotype association scan results for eye color. Dashed red lines correspond to the genome-wide significance threshold. (TIF) [file pgen.1003372.s001.tif]

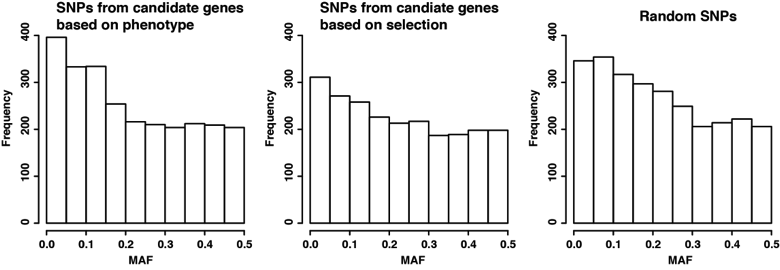

Supplement: Figure S2 — Distribution of allele frequencies for the two sets of candidate gene P values, and a control set of random SNPs (as depicted in Figure 5b). MAF, minor allele frequency. The distribution of the SNPs from the phenotype-based candidate genes is significantly different from the other two sets (P values for Kolmogorov-Smirnov tests are 0.01538 and 0.00944 for the comparison to selection-based candidate genes and random SNPs, respectively). The distribution of the SNPs from the selection-based candidate genes is not significantly different from the random SNPs (P = 0.125). (TIF) [file pgen.1003372.s002.tif]

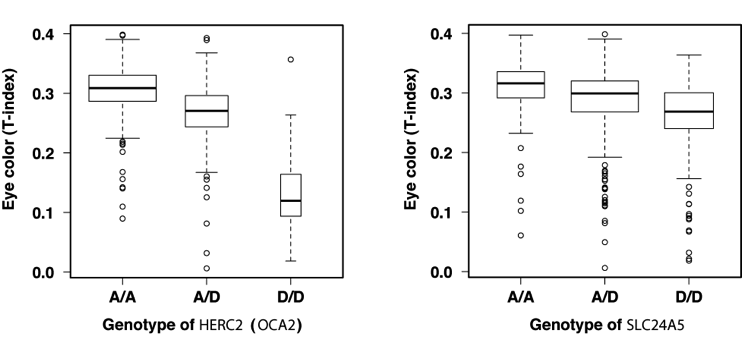

Supplement: Figure S3 — Effect of HERC2 (OCA2) and SLC24A5 genotype on eye color. Quantitative assessment of eye color with the T-index as described in the text and Figure 2. (TIF) [file pgen.1003372.s003.tif]
