# Supplementary material for: Genetic Architecture of Skin and Eye Color in an African-European Admixed Population
Source: PLoS Genet. 2013 Mar 21;9(3):e1003372. doi: 10.1371/journal.pgen.1003372 (PMC3605137; doi:10.1371/journal.pgen.1003372)
Supplement: Table S1 — GWAS results and derived allele frequencies stratified by island cluster. (DOCX) [file pgen.1003372.s004.docx]

**Table S1.** GWAS results and derived allele frequencies stratified by island cluster.

| **Chrom.**  **Region** | **Genes of interest in region** | **SNP** | **Santiago**  **(n= 172/141^b^)** | | |  | **Fogo**  **(n=129/122^b^)** | | |  | **Northern Islands ^a^**  **(n=262/ 250^b^)** | | |
| --- | --- | --- | --- | --- | --- | --- | --- | --- | --- | --- | --- | --- | --- |
|  |  |  | **Freq**^c^ | **Beta**^d^ | ***P*-value** |  | **Freq**^c^ | **Beta**^d^ | ***P*-value** |  | **Freq**^c^ | **Beta**^d^ | ***P*-value** |
| **Skin color** |  |  |  |  |  |  |  |  |  |  |  |  |  |
| 15q21.1 | *SLC24A5* | rs1426654 | 0.39 | -0.43 | 1.4 x10^-09^ |  | 0.62 | -0.30 | 8.2 x10^-04^ |  | 0.47 | -0.36 | 2.1 x10^-11^ |
| 11q14.3 | *GRM5, TYR* | rs10831496^e^ | 0.29 | -0.14 | 0.085 |  | 0.43 | -0.23 | 0.003 |  | 0.37 | -0.21 | 3.8 x10^-05^ |
| 15q13.1 | *APBA2* | rs4424881^e^ | 0.29 | -0.21 | 0.004 |  | 0.52 | -0.18 | 0.021 |  | 0.45 | -0.17 | 3.0 x10^-04^ |
| 5p13.3 | *SLC45A2* | rs35395^e^ | 0.31 | -0.18 | 0.019 |  | 0.47 | -0.13 | 0.118 |  | 0.42 | -0.25 | 9.6 x10^-07^ |
| Ancestry^f^ | *-* | - | - | -5.42 | 1.1 x10^-13^ |  | - | -5.50 | 1.7 x10^-09^ |  | - | -4.10 | 1.4 x10^-11^ |
| Ancestry^g^ (adjusted) | *-* | - | - | -3.93 | 3.5 x10^-09^ |  | - | -3.75 | 3.2 x10^-05^ |  | - | -2.46 | 2.9 x10^-06^ |
|  |  |  |  |  |  |  |  |  |  |  |  |  |  |
| **Eye color** |  |  |  |  |  |  |  |  |  |  |  |  |  |
| 15q13.1 | *HERC2, OCA2* | rs12913832 | 0.10 | -0.03 | 2.0x 10^-05^ |  | 0.26 | 0.047 | 3.7x10^-08^ |  | 0.20 | -0.07 | 1.5 x10^-38^ |
| 15q21.1 | *SLC24A5* | rs2470102^h^ | 0.40 | -0.01 | 0.003 |  | 0.62 | 0.018 | 0.023 |  | 0.47 | -0.02 | 3.1 x10^-08^ |

^a^ Barlavento, or the Northern Islands, refers to Santo Antão, São Vicente, São Nicolau and Boa Vista.

^b^ Number of individuals for skin/eye color GWAS.

^c^ Frequency of the derived, lightening, allele in each island.

^d^ Regression models includes the first three principal components and sex as covariates.

^e^ *P* values obtained after controlling for *SLC24A5*, in addition to the other covariates.

^f^ Estimated without any covariates.

^g^ Regression model includes genotype at the four major SNPs as covariates.

^h^ *P* values obtained after controlling for *HERC2*, in addition to the other covariates.
