# Supplementary material for: Genetic Architecture of Skin and Eye Color in an African-European Admixed Population
Source: PLoS Genet. 2013 Mar 21;9(3):e1003372. doi: 10.1371/journal.pgen.1003372 (PMC3605137; doi:10.1371/journal.pgen.1003372)
Supplement: Table S2 — Major loci for skin and eye color (without conditioning). (DOCX) [file pgen.1003372.s005.docx]

**Table S2.** Unadjusted *P* values for major skin and eye color loci

| **Chrom.  region** | **Candidate gene(s)** | **SNP** | **Significance ^a^** | |
| --- | --- | --- | --- | --- |
|  |  |  | **genotype** | **Ancestry** |
| **Skin color** |  |  |  |  |
| 15q21.1 | *SLC24A5* | rs1426654 | 5.4 x 10^-27^ | 3.9 x 10^-24^ |
| 11q14.3 | *GRM5-TYR* | rs10831496 **^b^** | 1.1 x 10^-09^ | 2.6 x 10^-07^ |
| 15q13.1 | *APBA2* | rs4424881 **^b^** | 1.5 x 10^-08^ | 2.7 x 10^-09^ |
| 5p13.3 | *SLC45A2* | rs35395 **^b^** | 1.3 x 10^-07^ | 7.3x 10^-06^ |
|  |  |  |  |  |
| **Eye color** |  |  |  |  |
| 15q13.1 | *HERC2,OCA2* | rs12913832 | 2.3 x 10^-62^ | 2.3 x10^-27^ |
| 15q21.1 | *SLC24A5* | rs2470102 **^b^** | 9. 6 x 10^-09^ | 5.1 x 10^-10^ |

^a^ *P* value for association of skin (n=685) or eye (n=625) color with genotype or local ancestry, adjusted for sex and the first three principal components, as described in Materials and Methods.

^b^ These entries differ from those reported in Table 1 in that there has been no adjustment for genotype at *SLC24A5* (skin) or *HERC2* (*OCA2*) (eye).
