# Supplementary material for: Genetic Architecture of Skin and Eye Color in an African-European Admixed Population
Source: PLoS Genet. 2013 Mar 21;9(3):e1003372. doi: 10.1371/journal.pgen.1003372 (PMC3605137; doi:10.1371/journal.pgen.1003372)
Supplement: Table S3 — Frequency of the derived allele for the eye and skin color loci at 15q13 in the Old World populations of HGDP and HapMap phase III datasets. (DOCX) [file pgen.1003372.s006.docx]

**Table S3.** Frequency of the derived allele for the eye and skin color loci at 15q13 in the Old World populations of HapMapIII and HGDP datasets.

|  | **Eye Color** |  | **Skin color** |
| --- | --- | --- | --- |
| **Population**^a^ | ***HERC2-*rs12913832** |  | ***APBA2-*rs4424881** |
| **Africa** |  |  |  |
| Bantu NE | 0.000 |  | 0.000 |
| Bantu SE | 0.000 |  | 0.000 |
| Biaka Pygmies | 0.000 |  | 0.031 |
| LWK | n.d^b^ |  | 0.050 |
| Mandenka | 0.000 |  | 0.167 |
| MKK | 0.000 |  | n.d^b^ |
| Mbuti Pygmies | 0.000 |  | 0.000 |
| Mozabite | 0.100 |  | 0.717 |
| San | 0.000 |  | 0.083 |
| YRI+Yoruba | 0.000 |  | 0.044 |
| **Western Asia** |  |  |  |
| Bedouin | 0.104 |  | 0.813 |
| Druze | 0.340 |  | 0.872 |
| Palestinian | 0.206 |  | 0.843 |
| **Central & South Asia** |  |  |  |
| Balochi | 0.180 |  | 0.740 |
| Brahui | 0.040 |  | 0.800 |
| Burusho | 0.300 |  | 0.640 |
| Hazara | 0.042 |  | 0.708 |
| Kalash | 0.280 |  | 0.640 |
| Makrani | 0.100 |  | 0.800 |
| Pathan | 0.196 |  | 0.783 |
| Sindhi | 0.060 |  | 0.720 |
| Uygur | 0.100 |  | 0.650 |
| **East Asia** |  |  |  |
| Cambodian | 0.000 |  | 0.455 |
| Dai | 0.000 |  | 0.400 |
| Daur | 0.000 |  | 0.389 |
| Han+CHB | 0.000 |  | 0.392 |
| Hezhen | 0.000 |  | 0.450 |
| Japanese+JPT | 0.000 |  | 0.496 |
| Lahu | 0.000 |  | 0.550 |
| Miaozu | 0.000 |  | 0.450 |
| Mongola | 0.000 |  | 0.600 |
| Naxi | 0.000 |  | 0.722 |
| Oroqen | 0.000 |  | 0.450 |
| She | 0.000 |  | 0.400 |
| Tu | 0.000 |  | 0.550 |
| Tujia | 0.000 |  | 0.450 |
| Xibo | 0.000 |  | 0.450 |
| Yizu | 0.000 |  | 0.600 |
| **Europe** |  |  |  |
| Adygei | 0.324 |  | 0.790 |
| CEU | 0.792 |  | 0.882 |
| French | 0.621 |  | 0.776 |
| French Basque | 0.396 |  | 0.792 |
| North Italian | 0.692 |  | 0.885 |
| Orcadian | 0.444 |  | 0.781 |
| Russian | 0.820 |  | 0.940 |
| Sardinian | 0.196 |  | 0.929 |
| TSI+Tuscan | 0.436 |  | 0.891 |
| **Oceania** |  |  |  |
| Melanesian | 0.000 |  | 0.421 |
| Yakut | 0.040 |  | 0.580 |
| Papuan | 0.000 |  | 0.500 |

^a^ HapMap population samples are denoted as on the website (www.hapmap.org). LWK: Luhya in Webuye, Kenya, MKK: Maasai in Kinyawa, Kenya, YRI: Yoruba in Ibadan, Nigeria, CEU: Utah residents with Northern and Western European ancestry from the CEPH collection, TSI: Toscans in Italy, CHB: Han Chinese in Beijing, China, JPT: Japanese in Tokyo, Japan. Samples from HGDP and HapMap datasets that come from the same population were added together for frequency estimation.

^b^ n.d, not determined.
