# Supplementary material for: Genetic Architecture of Skin and Eye Color in an African-European Admixed Population
Source: PLoS Genet. 2013 Mar 21;9(3):e1003372. doi: 10.1371/journal.pgen.1003372 (PMC3605137; doi:10.1371/journal.pgen.1003372)
Supplement: Table S4 — Candidate loci chosen based on function. (DOCX) [file pgen.1003372.s007.docx]

**Table S4.** Candidate loci chosen based on function.

| **Gene^a^** | **Chr** | **Start (bp)^b^** | **End (bp)^b^** | **Size (Kb)^b^** |
| --- | --- | --- | --- | --- |
| **Melanocyte development, migration and survival** |  |  |  |  |
| *MCOLN3* | chr1 | 85,206,352 | 85,336,757 | 130.4 |
| *MITF* | chr3 | 69,821,322 | 70,150,177 | 328.9 |
| *KIT* | chr4 | 55,168,851 | 55,351,638 | 182.8 |
| *GNAQ* | chr9 | 79,475,010 | 79,886,012 | 411.0 |
| *KITLG* | chr12 | 87,360,697 | 87,548,369 | 187.7 |
| *EDNRB* | chr13 | 77,317,616 | 77,497,665 | 180.0 |
| *GNA11* | chr19 | 2,995,407 | 3,122,454 | 127.0 |
| *EDN3* | chr20 | 57,258,893 | 57,384,442 | 125.5 |
| *SOX18* | chr20 | 62,099,522 | 62,201,423 | 101.9 |
| *SOX10* | chr22 | 36,648,264 | 36,760,485 | 112.2 |
| **Pigment type-switching** |  |  |  |  |
| *POMC* | chr2 | 25,187,225 | 25,295,063 | 107.8 |
| *SLC7A11* | chr4 | 139,254,697 | 139,432,953 | 178.3 |
| *MC1R* | chr16 | 88,461,787 | 88,564,886 | 103.1 |
| *MGRN1* | chr16 | 4,564,826 | 4,730,975 | 166.1 |
| *ASIP* | chr20 | 32,261,831 | 32,370,809 | 109.0 |
| *ATRN* | chr20 | 3,349,664 | 3,629,769 | 280.1 |
| **Melanogenesis** |  |  |  |  |
| *GPNMB* | chr7 | 23,202,840 | 23,331,254 | 128.4 |
| *TYRP1* | chr9 | 12,633,385 | 12,750,266 | 116.9 |
| *RAB38* | chr11 | 87,436,078 | 87,598,247 | 162.2 |
| *SILV* | chr12 | 54,584,155 | 54,696,093 | 111.9 |
| *DCT* | chr13 | 93,839,841 | 93,979,937 | 140.1 |
| *OCA2* | chr15 | 25,623,615 | 26,068,053 | 444.4 |
| **Melanosome biogenesis** |  |  |  |  |
| *LYST* | chr1 | 233,840,968 | 234,146,843 | 305.9 |
| *HPS3* | chr3 | 150,280,060 | 150,423,995 | 143.9 |
| *CNO* | chr4 | 6,718,742 | 6,820,288 | 101.5 |
| *AP3B1 [HPS2]* | chr5 | 77,283,905 | 77,676,284 | 392.4 |
| *MUTED [MU]* | chr6 | 7,909,213 | 8,059,646 | 150.4 |
| *DTNBP1* | chr6 | 15,581,017 | 15,821,250 | 240.2 |
| *ATP7B* | chr13 | 51,354,805 | 51,533,631 | 178.8 |
| *HPS1* | chr10 | 100,115,945 | 100,246,694 | 130.7 |
| *HPS6* | chr10 | 103,765,136 | 103,867,783 | 102.6 |
| *BLOC1S2* | chr10 | 101,973,702 | 102,086,429 | 112.7 |
| *HPS5* | chr11 | 18,206,792 | 18,350,297 | 143.5 |
| *VPS33A* | chr12 | 121,232,046 | 121,367,021 | 135.0 |
| *BLOC1S1* | chr12 | 54,346,086 | 54,449,754 | 103.7 |
| *RABGGTA* | chr14 | 23,754,583 | 23,860,643 | 106.1 |
| *PLDN* | chr15 | 43,616,708 | 43,739,201 | 122.5 |
| *AP3D1* | chr19 | 2,001,992 | 2,152,556 | 150.6 |
| *BLOC1S3* | chr19 | 50,323,842 | 50,426,898 | 103.1 |
| *HPS4* | chr22 | 25,127,445 | 25,259,820 | 132.4 |
| **Melanosome transport** |  |  |  |  |
| *MLPH* | chr2 | 238,010,616 | 238,178,700 | 168.1 |
| *MREG* | chr2 | 216,465,558 | 216,636,591 | 171.0 |
| *MYO7A* | chr11 | 76,466,957 | 76,653,934 | 187.0 |
| *MYO5A* | chr15 | 50,336,774 | 50,658,539 | 321.8 |
| *RAB27A* | chr15 | 53,233,091 | 53,419,293 | 186.2 |
| **Response to DNA damage** |  |  |  |  |
| *TP53* | chr17 | 7,462,444 | 7,581,588 | 119.1 |
| *ERCC2* | chr19 | 50,496,488 | 50,615,685 | 119.2 |
| **Associated with pigmentation phenotypes in GWAS studies** |  |  |  |  |
| *IRF4* | chr6 | 236,738 | 406,443 | 119.7 |
| *SLC24A4* | chr14 | 91,808,677 | 92,087,578 | 278.9 |

^a^ Genes with a potential role in human skin color based on their phenotypes in model organisms, or in humans affected with albinism.

^b^ Start, end, and size of the genomic regions collected for analysis, corresponding to the gene ± 50 Kb.
