# Supplementary material for: Genetic Architecture of Skin and Eye Color in an African-European Admixed Population
Source: PLoS Genet. 2013 Mar 21;9(3):e1003372. doi: 10.1371/journal.pgen.1003372 (PMC3605137; doi:10.1371/journal.pgen.1003372)
Supplement: Table S5 — Candidate loci chosen based on selective signatures. (DOCX) [file pgen.1003372.s008.docx]

**Table S5.** Candidate loci chosen based on selective signatures.

| **Region** | **Genes in Region** | **Chr** | **Start (bp)^a^** | **End (bp)^a^** | **Size (Kb)^a^** |
| --- | --- | --- | --- | --- | --- |
| **I** | *ZMYM6* | chr1 | 35,224,353 | 35,270,156 | 45.8 |
| **II** | *DARC* | chr1 | 157,440,426 | 157,442,914 | 2.5 |
| **III** | *ZRANB3, LCT, R3HDM1, MCM6* | chr2 | 135,808,531 | 136,350,481 | 516.6 |
| **IV** | *NFE2L2, AGPS* | chr2 | 177,803,278 | 178,116,810 | 313.5 |
| **V** | *RARB, TOP2B, NGLY1, OXSM* | chr3 | 25,444,757 | 25,811,029 | 366.3 |
| **VI** | *NAALADL2* | chr3 | 176,059,804 | 177,006,122 | 946.3 |
| **VII** | *CTNND2* | chr5 | 11,738,818 | 11,893,734 | 154.9 |
| **VIII** | *KCND2* | chr7 | 119,700,957 | 120,177,623 | 476.7 |
| **IX** | *SPIN1* | chr9 | 90,193,116 | 90,283,442 | 90.3 |
| **X** | *COMMD3, BMI1, SPAG6* | chr10 | 22,645,317 | 22,746,545 | 101.2 |
| **XI** | *PRKCH* | chr14 | 60,858,267 | 61,087,451 | 229.2 |
| **XII** | *KCNH5, RHOJ, GPHB5, PPP2R5E, WDR89* | chr14 | 62,243,697 | 63,178,344 | 934. 7 |
| **XIII** | *WWOX* | chr16 | 77,061,737 | 77,089,133 | 27.4 |
| **XIV** | *USP32, C17orf64, APPBP2, PPM1D, BCAS3* | chr17 | 55,609,472 | 56,824,981 | 1215.5 |
| **XV** | *RGS9, AXIN2* | chr17 | 60,563,917 | 60,988,202 | 424.3 |
| **XVI** | *C21orf34* | chr21 | 16,364,713 | 16,901,413 | 536.7 |

^a^ Start, end, and size of the genomic regions collected for analysis.
